# Endoscopic submucosal dissection using scissors-type knife for a giant solitary duodenal polyp

**DOI:** 10.1016/j.vgie.2021.04.011

**Published:** 2021-05-28

**Authors:** Michael Bejjani, Muhammad Nadeem Yousaf, Bachir Ghandour, Marcia Irene Canto, Mouen Khashab

**Affiliations:** 1 Johns Hopkins Hospital, Baltimore, Maryland; 2 Medstar Union Memorial Hospital, Baltimore, Maryland

**Keywords:** ESD, endoscopic submucosal dissection, PJ, Peutz-Jeghers

## Abstract

Video 1

## Background

Resection of giant pedunculated duodenal polyps is challenging. En bloc resection is preferred because of the risk of invasive cancer. EMR carries a significant risk of bleeding and perforation and may not be feasible when polyps are large. Traditional endoscopic submucosal dissection (ESD) carries a significant risk of perforation owing to the thin muscular layer of the duodenal wall and poor endoscopic operability. A scissors-type ESD knife has been increasingly used for resection of polyps in the stomach, duodenum, and colon.^1^, ^2^, ^3^, ^4^ In our case, we demonstrate an ESD procedure using the standard scissors-type knife for a giant duodenal polyp.

## Case presentation

**Figure 1:**
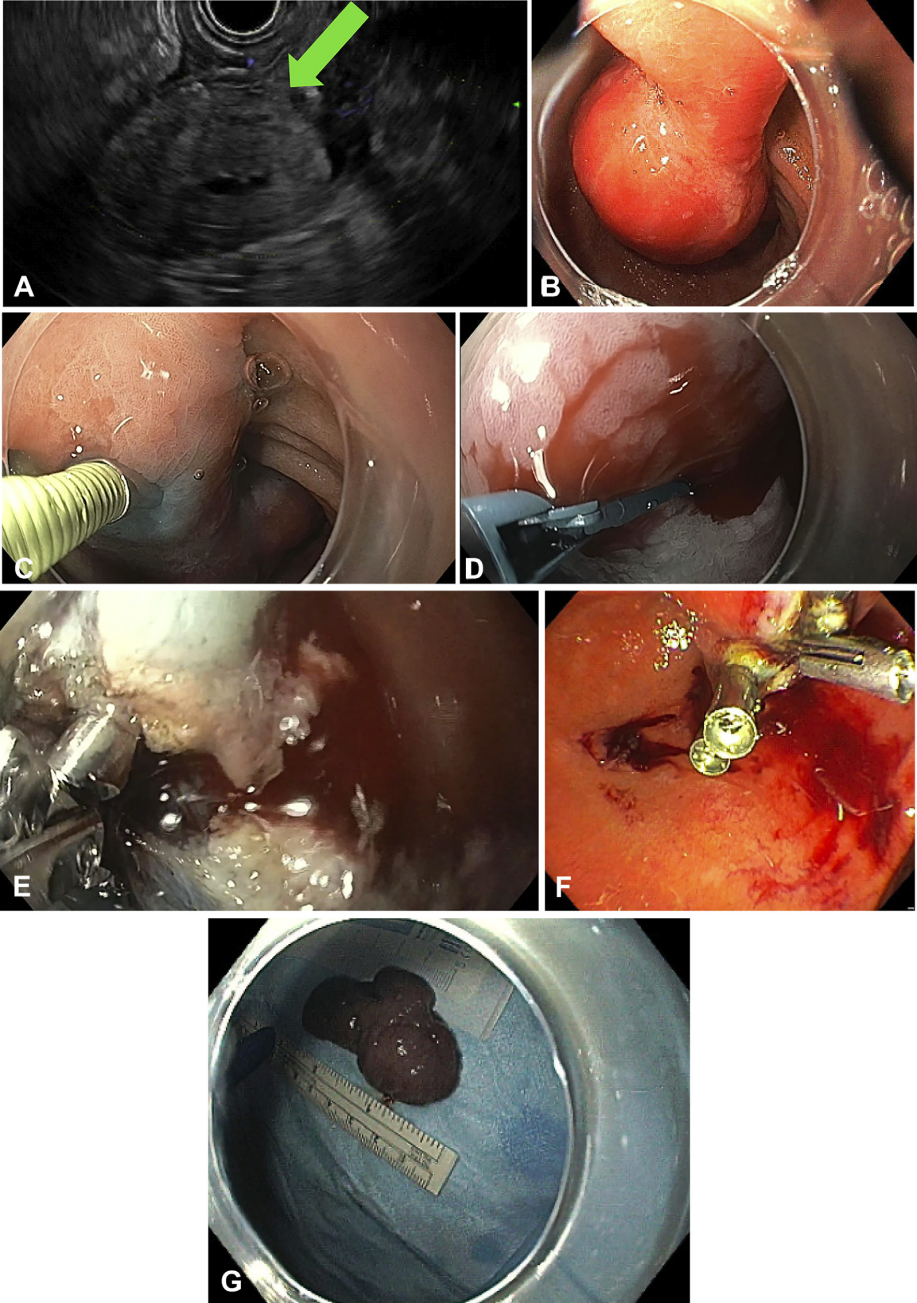
**A,** Duodenal polyp seen under EUS. **B,** Endoscopic view of duodenal polyp with stalk. **C,** Submucosal injection. **D,** Mucosal incision. **E,** Coagulation of bleeding vessel with coagulation forceps. **F,** Endoscopic closure with through-the scope clips. **G,** Polyp after resection and retrieval.

A 74-year-old man presented with a 1-year history of early satiety and feeling of epigastric discomfort after eating. EGD revealed a 6-cm pedunculated polyp originating from the duodenal bulb. EUS-FNA was performed, and cytopathology showed rare clusters of spindle cells suggestive of a leiomyoma (Fig. 1A). After a few months, the patient’s symptoms worsened and he was referred for definitive endoscopic therapy. The patient underwent en bloc polyp resection using a standard scissors-type knife.

## Video description

EGD was performed using a high-definition gastroscope fitted with a clear cap and advanced to the first part of the duodenum (Video 1, available online at www.giejournal.org). A giant pedunculated polyp with a large stalk was seen arising from the duodenal bulb and extending to the second duodenum (Fig. 1B). The pylorus muscle was injected with 100 units of botulinum toxin to ensure relaxation and facilitate retrieval of the giant polyp.

A mixture of 6% hetastarch, methylene blue (5 mg/1 mL), and diluted epinephrine (1:100,000) was injected into the submucosa to elevate the lesion from the muscular layer (Fig. 1C). Dissection was then commenced using the standard scissors-type knife (7-mm forceps length and 8-mm opening width) using EndoCut Q current, effect 4. Dissection was started at the stalk several millimeters from the polyp margin (Fig. 1D). Similarly, dissection was performed away from the bottom of the stalk to avoid injury to the duodenal wall. The intention was to leave enough stalk to aid in easy closure with clips at the end of dissection.

During dissection, we encountered minor bleeding, which was controlled with coagulation using the ESD scissors knife (soft coagulation 80 W, effect 5) and a coagulation forceps (Fig. 1E). Submucosal dissection was continued until complete en bloc polyp resection was achieved. Clear resection margins with no remnant tissue were observed after dissection of the polyp. The polyp was then retrieved using a large forceps. When examined outside, the polyp measured around 6 cm in length (Fig 1G). Complete closure of the polypectomy site was achieved using a duodenoscope and 5 through-the-scope clips (Sure Clip, Micro-Tech; Ann Arbor, Mich, USA) (Fig. 1F). Procedure time was 45 minutes, with only minor bleeding that was quickly managed with coagulation.

## Outcomes

The patient was admitted overnight. One day after the procedure, the patient was able to tolerate peroral intake and was discharged on proton pump inhibitors. Pathology confirmed R0 resection of a 6-cm hamartomatous polyp. At 1-month follow-up, the patient was feeling well with complete resolution of his symptoms.

Solitary hamartomatous polyps were previously considered an incomplete or initial form of Peutz-Jeghers (PJ) syndrome. However, it was shown that not all cases express symptoms of PJ syndrome, such as mucocutaneous pigmentation or family history of intestinal polyps. Thus, solitary hamartomatous polyps now constitute a different entity than PJ syndrome, for which the definitive treatment is endoscopic resection.^5^

## Conclusions

Our case shows that the scissors-type ESD knife enables easy, efficient, and safe en bloc resection of giant pedunculated duodenal polyps. This technique should be increasingly considered for the resection of such polyps because it enables efficient and safe resection.

## Disclosure

*Dr Canto has received grants from Pentax Medical Corporation and Endogastric Solutions and receives royalties from UpToDate. Dr Khashab is a consultant for Boston Scientific, Olympus, Medtronic, and GI Supply and receives royalties from Udtodate and Elsevier. All other authors disclosed no financial relationships.*

## Supplementary data

Video 1 Endoscopic submucosal dissection using scissors-type knife for a giant solitary duodenal polyp.
